# Genome-wide association study of morbid obesity in Han Chinese

**DOI:** 10.1186/s12863-019-0797-x

**Published:** 2019-12-18

**Authors:** Kuang-Mao Chiang, Heng-Cheng Chang, Hsin-Chou Yang, Chien-Hsiun Chen, Hsin-Hung Chen, Wei-Jei Lee, Wen-Harn Pan

**Affiliations:** 1 Institute of Biomedical Sciences, Academia Sinica, Taipei City, Taiwan; 2 Department of Gynecology and Obstetrics, School of Medicine, Taipei Medical University, Taipei City, Taiwan; 3 Institute of Statistical Science, Academia Sinica, Taipei City, Taiwan; 4 Department of Nutrition and Health Science, Chang Jung Christian University, Tainan City, Taiwan; 5 Department of Surgery, Min-Sheng General Hospital, Taoyuan City, Taiwan; 6 Institute of Population Health Sciences, National Health Research Institutes, Miaoli County, Taiwan

**Keywords:** Morbid obesity, Body mass index, Genome-wide association study, FTO

## Abstract

**Background:**

As obesity is becoming pandemic, morbid obesity (MO), an extreme type of obesity, is an emerging issue worldwide. It is imperative to understand the factors responsible for huge weight gain in certain populations in the modern society. Very few genome-wide association studies (GWAS) have been conducted on MO patients. This study is the first MO-GWAS study in the Han-Chinese population in Asia.

**Methods:**

We conducted a two-stage GWAS with 1110 MO bariatric patients (body mass index [BMI] ≥ 35 kg/m^2^) from Min-Sheng General Hospital, Taiwan. The first stage involved 575 patients, and 1729 sex- and age-matched controls from the Taiwan Han Chinese Cell and Genome Bank. In the second stage, another 535 patients from the same hospital were genotyped for 52 single nucleotide polymorphisms (SNPs) discovered in the first stage, and 9145 matched controls from Taiwan Biobank were matched for confirmation analysis.

**Results:**

The results of the joint analysis for the second stage revealed six top ranking SNPs, including rs8050136 (*p*-value = 7.80 × 10^− 10^), rs9939609 (*p*-value = 1.32 × 10^− 9^), rs1421085 (*p*-value = 1.54 × 10^− 8^), rs9941349 (*p*-value = 9.05 × 10^− 8^), rs1121980 (*p*-value = 7.27 × 10^− 7^), and rs9937354 (*p*-value = 6.65 × 10^− 7^), which were all located in *FTO* gene. Significant associations were also observed between MO and *RBFOX1, RP11-638 L3.1, TMTC1, CBLN4, CSMD3*, and *ERBB4*, respectively, using the Bonferroni correction criteria for 52 SNPs (*p* < 9.6 × 10^− 4^).

**Conclusion:**

The most significantly associated locus of MO in the Han-Chinese population was the well-known *FTO* gene. These SNPs located in intron 1, may include the leptin receptor modulator. Other significant loci, showing weak associations with MO, also suggested the potential mechanism underlying the disorders with eating behaviors or brain/neural development.

## Background

Obesity is a chronic phenomenon of positive energy balance, leading to the long-term and excessive accumulation of body fat. Epidemiological studies have revealed the substantial increase in the risk of Non-Communicable Diseases (NCD) in people with morbid obesity (MO) [1].

The latest evidence indicates the sharp rise in the prevalence of MO worldwide in both men and women [2]. In the US, the prevalence MO has increased by more than four-fold (1.4 to 6.3%) within the last three decades [3]. Notably, the prevalence of MO (body mass index [BMI] ≥ 35 kg/m^2^) [4, 5] in Taiwan has also increased from almost null to 1.3% during the past two decades, as per the data collected by the Nutrition and Health Survey in Taiwan (NAHSIT) from 1993 to 1996 to 2013–2016 [4]. As MO is accompanied with multiple comorbidity [6, 7], including shorter life expectancy and higher all-cause mortality rate [7, 8] than that in general public, the associated medical cost and social economic burden are tremendous [9]. Lifestyle intervention measures are less efficient for MO cases, and bariatric surgery is expensive and could induce complications [10].

The Global Burden of Disease study has pointed out poor diet (western or super-processed) in combination with physical inactivity/sedentary lifestyles as the main risk factors of non-communicable diseases, including obesity, diabetes [11–14], and associated cardio-metabolic diseases. However, BMI distribution is very wide, indicative of the differences in individual responses to the same obesogenic environment. It is worthy to investigate the genetic mechanisms underlying the development of extreme cases of obesity [15–18].

According to twin, family, and adoption studies, the heritability of BMI is estimated to be around 40–70% [19–22], and approximately 27% of BMI heritability may be attributed to common single nucleotide polymorphism (SNP) in adults [23]. A review on genome-wide association studies (GWAS) has documented at least 741 BMI- or obesity-related SNPs and numerous biological pathways [24]. MO, as the extreme type of obesity, may be highly associated with the common BMI-raising variants [25, 26].

Several GWAS have been performed on severe obesity and MO [27–33]. However, some of these MO-GWAS involved children or adolescents with high BMI percentile values, and all included European populations. Our study is the first MO-GWAS conducted in Chinese population in the Asian region.

## Results

Additional file 1: Table S1 shows the comparison of the sample characteristics between MO patients at stage 1 and stage 2. No significant differences were observed between the two groups. Although some differences between the two control groups were noted, these differences (sex and age) were adjusted in the regression models.

### Two-stage GWAS

**Fig. 1:**
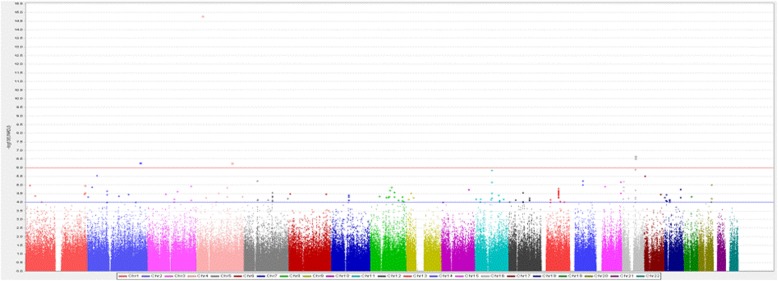
The Manhattan plots of the first stage of MO-GWAS. GWAS for MO was analyzed by logistic regression with age, sex and PC 1 to PC10 adjustment. Blue line: -log10 *p*-value = 4; Red line: -log10 *p*-value = 6

Figure 1 is the Manhattan plots of the 1st stage MO-GWAS. Additional file 1: Table S2 shows the 80 SNPs, with *p*-value < 10^− 4^ considered for 2nd stage confirmation. The SNP rs116917414 is the most significant SNP in the first stage GWAS (*p*-value = 1.15 × 10^− 12^). Sixteen SNPs were not used for further analysis due to differences in probe design between CHB-1 Array and TWB Array. Twelve SNPs, which showed poor genotyping quality (CR < 97%, MAF < 1%, or HWE < 0.001), were further removed. Finally, 52 SNPs were included in the 2nd stage. In the 2nd stage (Table 1), only one SNP, rs8050136 (*p*-value = 9.3 × 10^− 4^), was significant following the Bonferroni correction (*p*-value < 9.6 × 10^− 4^ [0.05/52]).

**Table 1:** The two-stage MO-GWAS and joint analysis results of 52 SNPs

CHR	SNP	POSITION	Gene (Nearest)	Minor Allele	1st Stage			2nd Stage			Joint analysis (1st + 2nd)			
					OR	SE	Raw-*p*	OR	SE	Raw-*p*	OR	SE	Raw-*p*	Bonferroni (Raw-*p* × 52)
16	rs8050136	53,816,275	FTO	A	1.73	0.11	2.11E-07	1.37	0.09	9.37E-04	1.51	0.07	***7.80E-10***^***$***^	4.13E-08*
16	rs9939609	53,820,527	FTO	A	1.73	0.11	2.20E-07	1.35	0.10	1.58E-03	1.51	0.07	***1.32E-09***^***$***^	7.00E-08*
16	rs1421085	53,800,954	FTO	C	1.65	0.10	1.31E-06	1.32	0.09	3.18E-03	1.46	0.07	***1.54E-08***^***$***^	8.16E-07*
16	rs9941349	53,825,488	FTO	T	1.62	0.09	2.82E-07	1.42	0.18	4.83E-02	1.48	0.07	9.05E-08	4.80E-06*
16	rs1121980	53,809,247	FTO	A	1.59	0.09	1.16E-06	1.19	0.09	5.35E-02	1.36	0.06	7.27E-07	3.85E-05*
16	rs9937354	53,799,847	FTO	A	1.57	0.09	1.18E-06	1.38	0.18	7.46E-02	1.43	0.07	6.65E-07	3.52E-05*
16	rs12925846	6,926,667	RBFOX1	G	0.68	0.09	6.04E-06	0.87	0.08	6.68E-02	0.78	0.06	6.21E-06	3.29E-04*
16	rs17235335	6,928,991	RBFOX1	G	0.69	0.09	1.30E-05	0.88	0.08	8.06E-02	0.79	0.05	1.26E-05	6.68E-04*
14	rs11626956	51,850,031	N/A	G	1.40	0.08	9.57E-06	1.09	0.07	2.39E-01	1.23	0.05	3.35E-05	0.0018*
18	rs2126015	65,545,054	RP11-638 L3.1	C	0.70	0.08	1.76E-05	0.90	0.07	1.69E-01	0.81	0.05	4.64E-05	0.0025*
12	rs159702	29,748,233	TMTC1	C	1.44	0.09	7.31E-05	1.15	0.08	8.60E-02	1.25	0.06	1.50E-04	0.0080*
20	rs6069477	54,491,980	CBLN4	A	1.40	0.08	9.40E-06	1.05	0.07	5.02E-01	1.20	0.05	2.87E-04	0.015*
8	rs16883931	113,664,153	CSMD3	G	1.36	0.08	7.40E-05	1.12	0.07	1.22E-01	1.20	0.05	3.99E-04	0.021*
2	rs6757087	212,680,523	ERBB4	G	1.46	0.08	5.19E-07	1.00	0.07	9.68E-01	1.19	0.05	5.49E-04	0.029*
2	rs9808434	18,694,885	N/A	C	1.45	0.09	1.29E-05	1.05	0.08	5.59E-01	1.20	0.06	8.83E-04	0.047*
8	rs72621457	64,548,431	LOC102724612	G	0.70	0.09	5.12E-05	0.96	0.08	5.64E-01	0.83	0.06	1.12E-03	0.059
4	rs12649045	122,957,607	TRPC3, KIAA1109	T	0.71	0.08	1.40E-05	1.03	0.07	7.12E-01	0.85	0.05	1.31E-03	0.069
8	rs3104994	96,571,283	LOC100616530, C8orf37-AS1	C	1.54	0.11	4.84E-05	1.03	0.10	7.43E-01	1.24	0.07	1.84E-03	0.098
5	rs258019	115,807,688	CTB-118 N6.3 / SEMA6A	C	0.74	0.08	8.26E-05	0.98	0.07	7.29E-01	0.86	0.05	2.39E-03	0.13
1	rs3003482	17,550,601	PADI1	G	0.70	0.08	1.02E-05	1.00	0.07	9.65E-01	0.86	0.05	3.01E-03	0.16
18	rs76010193	65,526,327	RP11-638 L3.1	T	0.73	0.08	5.21E-05	0.98	0.07	7.54E-01	0.86	0.05	2.89E-03	0.15
9	rs74596508	89,361,079	LOC102724080, GAS1	G	0.60	0.14	2.71E-04	0.95	0.26	8.38E-01	0.71	0.12	3.19E-03	0.17
3	rs4859033	88,518,323	N/A	G	0.73	0.08	2.03E-04	0.93	0.08	3.67E-01	0.85	0.06	3.85E-03	0.20
5	rs34971	115,795,188	CTB-118 N6.3 / SEMA6A	T	1.36	0.08	4.25E-05	0.99	0.07	8.63E-01	1.15	0.05	4.34E-03	0.23
12	rs7136269	58,075,937	RPL13AP23 (Pseudo), OS9	A	0.73	0.08	9.66E-05	1.02	0.15	9.19E-01	0.84	0.06	4.71E-03	0.25
4	rs985591	63,331,949	N/A	T	1.39	0.09	1.28E-04	1.02	0.08	8.37E-01	1.17	0.06	5.22E-03	0.28
13	rs9571975	69,218,765	RPS3AP52 (Pseudo), RPL12P34 (Pseudo)	A	1.47	0.09	3.02E-05	0.94	0.09	4.81E-01	1.19	0.06	5.75E-03	0.30
5	rs79393925	80,873,159	SSBP2	C	0.46	0.21	1.47E-04	0.76	0.21	1.86E-01	0.67	0.15	5.84E-03	0.31
12	rs10876994	58,064,737	RPL13AP23 (Pseudo)	A	0.75	0.08	1.60E-04	1.12	0.15	4.59E-01	0.85	0.06	6.15E-03	0.33
11	rs2575826	14,474,971	N/A	T	0.63	0.12	2.00E-04	0.98	0.11	8.69E-01	0.81	0.08	6.64E-03	0.35
16	rs205186	25,574,869	N/A	T	1.52	0.12	4.98E-04	1.05	0.11	6.67E-01	1.24	0.08	7.49E-03	0.40
3	rs59778278	120,809,596	STXBP5L	A	0.53	0.15	2.31E-05	0.89	0.14	4.00E-01	0.77	0.10	1.04E-02	0.55
16	rs237140	26,645,648	N/A	C	0.54	0.15	5.79E-05	1.07	0.12	5.95E-01	0.80	0.09	1.51E-02	0.80
18	rs17681801	50,173,621	DCC	C	1.35	0.08	1.92E-04	1.02	0.08	8.18E-01	1.13	0.05	1.90E-02	1.01
3	rs117383450	168,549,587	N/A	A	0.47	0.21	3.98E-04	0.96	0.17	8.09E-01	0.74	0.13	2.11E-02	1.12
8	rs3104999	96,555,479	LOC100616530, C8orf37-AS1	A	1.33	0.08	2.73E-04	1.00	0.07	9.49E-01	1.12	0.05	2.35E-02	1.25
15	rs16959465	33,129,842	FMN1	G	0.75	0.08	3.58E-04	1.03	0.07	6.64E-01	0.89	0.05	2.37E-02	1.26
1	rs351608	4,384,773	N/A	G	0.72	0.10	4.45E-04	0.94	0.19	7.42E-01	0.84	0.08	2.32E-02	1.23
4	rs2350064	66,709,201	N/A	T	1.50	0.11	1.71E-04	1.00	0.10	9.76E-01	1.17	0.07	2.48E-02	1.31
17	rs465563	3,513,719	SHPK / TRPV1	G	1.46	0.08	3.04E-06	0.91	0.07	2.06E-01	1.12	0.05	2.92E-02	1.55
8	rs10106743	52,371,648	PXDNL	T	0.58	0.16	4.85E-04	0.99	0.30	9.77E-01	0.76	0.13	3.10E-02	1.64
12	rs4763799	12,462,567	LRP6, MANSC1	C	0.71	0.09	1.64E-04	1.05	0.08	5.34E-01	0.88	0.06	3.23E-02	1.71
13	rs9571961	69,167,161	LOC102724158	T	1.48	0.09	2.05E-05	0.88	0.09	1.78E-01	1.14	0.06	3.57E-02	1.89
9	rs2082632	8,794,253	PTPRD	G	0.75	0.08	4.63E-04	1.05	0.07	5.09E-01	0.90	0.05	4.30E-02	2.28
8	rs72664169	90,126,780	N/A	A	1.34	0.08	1.25E-04	0.94	0.07	3.84E-01	1.10	0.05	5.21E-02	2.76
12	rs61919537	63,058,514	PPM1H	C	0.62	0.13	1.35E-04	1.10	0.10	3.72E-01	0.86	0.08	5.90E-02	3.13
1	rs351607	4,384,296	RP5-1166F10.1 (Pseudo)	G	0.70	0.10	2.30E-04	1.03	0.09	7.23E-01	0.89	0.06	6.56E-02	3.48
5	rs17741008	150,896,898	FAT2 / SLC36A1	T	0.71	0.10	4.30E-04	1.08	0.08	3.89E-01	0.90	0.06	7.45E-02	3.95
2	rs2892923	165,438,473	GRB14	C	1.67	0.12	3.38E-05	0.77	0.13	5.04E-02	1.13	0.08	1.57E-01	8.32
1	rs4259589	69,972,693	N/A	G	0.75	0.08	4.45E-04	1.16	0.07	3.88E-02	0.93	0.05	1.90E-01	10.07
6	rs6907980	18,401,381	RNF144B	A	1.35	0.08	3.07E-04	0.88	0.08	1.15E-01	1.06	0.05	2.76E-01	14.63
3	rs1449907	22,573,833	N/A	G	0.73	0.09	2.61E-04	1.13	0.07	9.45E-02	0.95	0.05	3.04E-01	16.11

CHR chromosome, SE standard error, Raw-p: raw p-value. $: *p*-value < 5 × 10^−8^; *: Bonferroni correction *p*-value (raw-p x 52) <  0.05

### Joint analyses

Table 1 shows the results of the joint analyses for the 52 SNPs in 1110 MO patients and 10,852 matched controls. Among these 52 SNPs, rs8050136 (*p*-value = 7.80 × 10^− 10^), rs9939609 (*p*-value = 1.32 × 10^− 9^), rs1421085 (*p*-value = 1.54 × 10^− 8^), rs9941349 (*p*-value = 9.05 × 10^− 8^), rs1121980 (*p*-value = 7.27 × 10^− 7^), and rs9937354 (*p*-value = 6.65 × 10^− 7^) were the top ranking SNPs, and all located in the same linkage disequilibrium (LD) block (Additional file 1: Figure S1) in the intron 1 of *FTO* gene. Nine additional SNPs showed statistical significance using the Bonferroni correction criteria (p-value < 9.6 × 10^− 4^ [0.05/52]). Seven SNPs flanked six loci as follows: *RBFOX1* (rs12925846 [*p*-value = 6.21 × 10^− 6^], and rs17235335 [*p*-value = 1.26 × 10^− 5^]), *RP11-638 L3.1* (rs2126015, *p*-value = 1.26 × 10^− 5^), *TMTC1* (rs159702, *p*-value = 1.26 × 10^− 5^), *CBLN4* (rs6069477, *p*-value = 1.26 × 10^− 5^), *CSMD3* (rs16883931, *p*-value = 1.26 × 10^− 5^), and *ERBB4* (rs6757087, *p*-value = 1.26 × 10^− 5^). Two SNPs, rs11626956 (*p*-value = 1.26 × 10^− 5^), and rs9808434 (*p*-value = 1.26 × 10^− 5^) were located in an intergenic region.

## Discussion

This is the first MO-GWAS conducted using the Han-Chinese population in Asia. This GWAS, with 1110 MO patients and 10,852 matched controls in Han-Chinese population, established that the top 6 SNPs (rs8050136, rs9939609, rs1421085, rs9941349, rs1121980, and rs9937354) were all located in the most replicable obesity gene: the *FTO*.

In 2007, the well-known obesity gene, *FTO*, was first identified in a European ancestry population [34]. Since then, *FTO* has been replicated and validated in many other ethnic populations, including African [35] and Asian [36] populations. The association between *FTO* and severe obesity or MO is also reported in the European [37] and Japanese [38] populations. However, the evidence has been very limited for Han-Chinese, the largest population in the world.

In this two-stage GWAS, we found that six SNPs on *FTO* top all SNPs in association with morbid obesity in Han-Chinese (rs8050136, rs9939609, rs1421085, rs9941349, rs1121980, and rs9937354), especially with the rs8050136 and rs9939609 and rs1421085 reaching *p* ≤ 5 × 10^− 8^. According to our data and HapMap data, these six SNPs are within the same LD block in the intron 1 of *FTO* gene (Additional file 1: Figure S1). Of these, rs9941349 was found to be associated with obesity for the first time.

The latest evidence indicated the association between rs9939609 of *FTO* (*p* = 0.026) and obesity (BMI ≥ 30 kg/m^2^) in 1188 Taiwanese subjects [39]. A previous meta-analysis study with 4189 Han-Chinese individuals also validated the association between obesity (BMI ≥ 28 kg/m^2^) and rs9939609 (odds ratio [OR]: 1.39, *p*-value = 0.02) along with rs8050136 (OR: 1.45, p-value = 0.01) [40]. In addition, the association between rs8050136 and obesity (BMI ≥ 27.5 kg/m^2^) is implicated in 1170 Chinese subjects [41], and rs1121980 has been replicated in Han-Chinese [42] and Malay populations [43]. Furthermore, rs1421085 is detectable in Chinese children aged 3 to 6 years [44].

Although rs9939609 is the most replicable *FTO* SNP, it is more prevalent in the European [45] populations (42%), than in Africans (12%) [46], East Asians (12–20%), and South Asians (30–33%) [39]. In our study, the MAF of rs9939609 was only 13.2%.

Claussnitzer et al. [47] suggested that rs1421085 may be the causal variant, instead of rs9939609 on *FTO* gene, as a single nucleotide variant alteration in rs1421085 (T-to-C) may cause disruption from the *ARID5B*-mediated suppression of *IRX3* and *IRX5*, leading to adipocyte developmental shift from browning (energy expenditure) to whitening (energy storage), and suppression of mitochondrial thermogenesis.

The SNPs rs8050136, rs9937354, rs1421085, and rs1121980, in the first intron of *FTO*, are located in an enhancer region. Recent studies have indicated that the links between the intronic variance within *FTO* and body composition are mediated through functional interactions with neighboring genes. The first intron of *FTO* carries a binding site for the transcription factor *CUX1*, which modulates the leptin receptor localization within neurons, through the regulation of *RPGRIP1L* expression. This intron also contains an enhancer sequence that directly binds to the promoter of *IRX3* [48, 49]. Therefore, the mechanisms underlying the contribution of *FTO* to the risk of obesity are apparently more complex than expected.

Aside from the *FTO*-related SNPs, nine SNPs were statistically significant according to the Bonferroni correction criteria, with *p*-value < 9.6 × 10^− 4^ (0.05/52) in the joint analysis. These SNPs flank *RBFOX1*, *RP11-638 L3.1, TMTC1, CBLN4, CSMD3 a*nd *ERBB4* genes*.*

Two significant SNPs of *RBFOX1* gene (RNA-binding fox-1 homolog 1) were discovered in this study, rs12925846 and rs17235335. This gene has been associated with several complex diseases, including schizophrenia, autism, mental retardation in epilepsy, attention deficit disorder, and obesity [50]. *RBFOX1* is thought to affect adiposity through the hypothalamic melanocortin 4 receptor (*MC4R*) pathway [51]. Mutations of *MC4R* are known to cause a monogenic form of obesity in humans [52] via leptin. In the brain, the hypothalamus is known as the control center for satiety/hunger and social defeat. *RBFOX1* gene, also known as ataxin-2-binding protein 1 gene (*A2BP1*), could regulate neuron-specific splicing by binding to the pentanucleotide (U) GCAUG sequences upstream of the regulated exon [53]. The involvement of *RBFOX1* in obesity development is questionable and warrants further investigation.

One MO-associated SNP, rs2126015, is located on the *RP11-638 L3.1* gene, a long noncoding RNA. Previous studies indicated the association of this SNP with neurological disorders such as attention deficit hyperactivity disorder (ADHD), and early-onset recurrent major depressive disorder (MDD) [54]. This gene is also highly expressed in the adipose tissue. lncRNAs are known to play important epigenetic regulatory roles in some important molecular processes, such as gene expression, genetic imprinting, histone modification, chromatin dynamics, and other activities, including formation of specific structures and interactions with all kinds of molecules [55]. The involvement of epigenetic modifications in the development of obesity is becoming increasingly evident [56, 57]. Obesity is associated with environmental pollutants (obesogens) [58], gut microbiota [59], and unbalanced food intake, all of which may result in weight gain, and altered metabolic consequences through epigenetic mechanisms. Further studies with a larger sample size are warranted to examine the interactions between genes and environmental factors, particularly dietary factors.

The gene *TMTC1* (rs159702) has been associated with heart failure in an African ancestry population [60]. Moreover, the interaction of *TMTC1* with abdominal obesity may contribute to phenotypic variation of left ventricular mass (LVM) [61]. However, the mechanism of *TMTC1* involvement in MO remains unclear.

The proteins encoded by gene *CBLN4* (rs6069477) are involved in the regulation of neurexin signaling during synapse development. Agouti related protein (*AGRP*)-expressing neurons are a key starvation-sensitive hypothalamic population, activated during energy deficit and increases appetite and weight gain. An animal study has shown that, *CBLN4* is downregulated in AGRP neurons after food-deprivation [62]. It is worth further investigating the mechanism between this gene and MO.

The rs16883931 is located in the *CSMD3* (CUB and Sushi Multiple Domains 3). This gene is a large protein expressed in the fetal and adult brain and is involved in dendrite development. Mutations of the *CSMD3* gene were identified in schizophrenic and autistic patients. However, biochemical properties and functions of the *CSMD3* protein remain unknown [63].

Another MO-associated gene *ERBB4* (rs29944391) is a member of the EGF receptor family. Genetic studies have indicated a link between *ERBB4* and type 2 diabetes, and obesity. Neuroregulin 4 (NRG4), a ligand that specifically binds to *ERBB4*, has been reported to promote browning of white fat, fuel oxidation, prevention of high-fat diet-induced obesity, and improvement of insulin sensitivity [64].

The SNP rs116917414 was the most significant SNP in the first stage GWAS (*p*-value = 1.15 × 10^− 12^). However, this SNP was not included in the second stage owing to the failure in probe design. While we searched for a proxy SNP for rs116917414 using 1000 Genome database, we were unable to detect any SNP in strong LD (r^2^ > 0.8) with rs116917414. Hence, we used the next-generation sequencing data (*N* = 1445) collected from the Taiwan Biobank to investigate the association between rs116917414 and BMI. No significant association was found between this SNP and BMI (*p*_GA vs. GG_ = 0.6, *p*_AA vs. GG_ = 0.5) (Additional file 1: Table S4), indicating the necessity for a larger sample size to confirm its effects. This SNP resides in the conserved noncoding region close to the *RP11-380P13.1* (ENSG00000250137) pseudogene promoter 5′-region. Notably, a study using Framingham data has reported the location of rs2130928 in the *RP11-380P13.1* and its association with BMI (*p* = 0.0012) [65]. As only little is known about the *RP11-380P13.1*, it is worthy of further research.

A recent GWAS for BMI in the Japanese population identified 85 SNPs [66]. We have investigated the association of these SNPs in our Han-Chinese population. Only six of these SNPs could be replicated in our study population (*p* < 0.05) (Additional file 1: Table S3), probably owing to the differences in studied traits, designs, and populations, as one involves cross-sectional GWAS with BMI as a quantitative trait in the Japanese general population, and the other was a case-control GWAS study of Chinese MO.

As this is the first large-scale MO-GWAS performed in the Han-Chinese population, the biological mechanisms or pathways known for some of the discovered genes are limited. Validation and mechanistic studies of these discovered genes are crucial. Patients with MO are those at the extreme tail of BMI distribution in population, within the same obesogenic environment. These patients show much higher increase in mean BMI in obesogenic environments, owing to genetic susceptibility [15–18]. A recent thought on the genetic underspin of the common complex traits is that “genes load the gun, but the environment pulls the trigger [67].” There were no obese individuals during famines, and the prevalence of obesity increased with increase in food supply. The subjects that present with greater genetic susceptibility to obesity are likely to gain more weight or fat in obesogenic environments. Individuals that carry the risk allele of *FTO* gene tend to have a higher protein [68] and calorie [69] intake. The interaction between genetic risk scores (from known obesity-related variants), and total fried food consumption and physical activity has been reported in NHS, HPFS, and Women’s Genome Health Study [70]. Moreover, the behavioral susceptibility theory has also suggested that genes control the response to food cues (smell, sight, and taste), and determine sensitivity to satiety in obesogenic environments [67].

## Conclusions

In summary, this is the first study illustrating genetic characteristics of MO in the Han-Chinese population. The most significantly associated locus of MO, in Han-Chinese population, was the well-known *FTO* gene. These SNPs, located in the intron 1, may include the leptin receptor modulator. In addition, other significant loci, including *RBFOX1, RP11-638 L3.1, TMTC1, CBLN4, CSMD3,* and *ERBB4*, showing weak associations with MO, suggested the potential mechanism underlying disorders with altered eating behaviors or brain/neural development, warranting further study on satiety control. Our results highlight the complexity of genetic involvement in the development of MO in humans.

## Methods

### Study design and sample size

We conducted a two-stage GWAS in Taiwan Han-Chinese population of 1110 patients with MO between 19 to 55 years of age. In total, 575 patients were included in the first stage and 535 patients, in the second stage. At the end, we carried out a joint analysis for those SNPs showing significant tendency in the first stage.

**Fig. 2:**
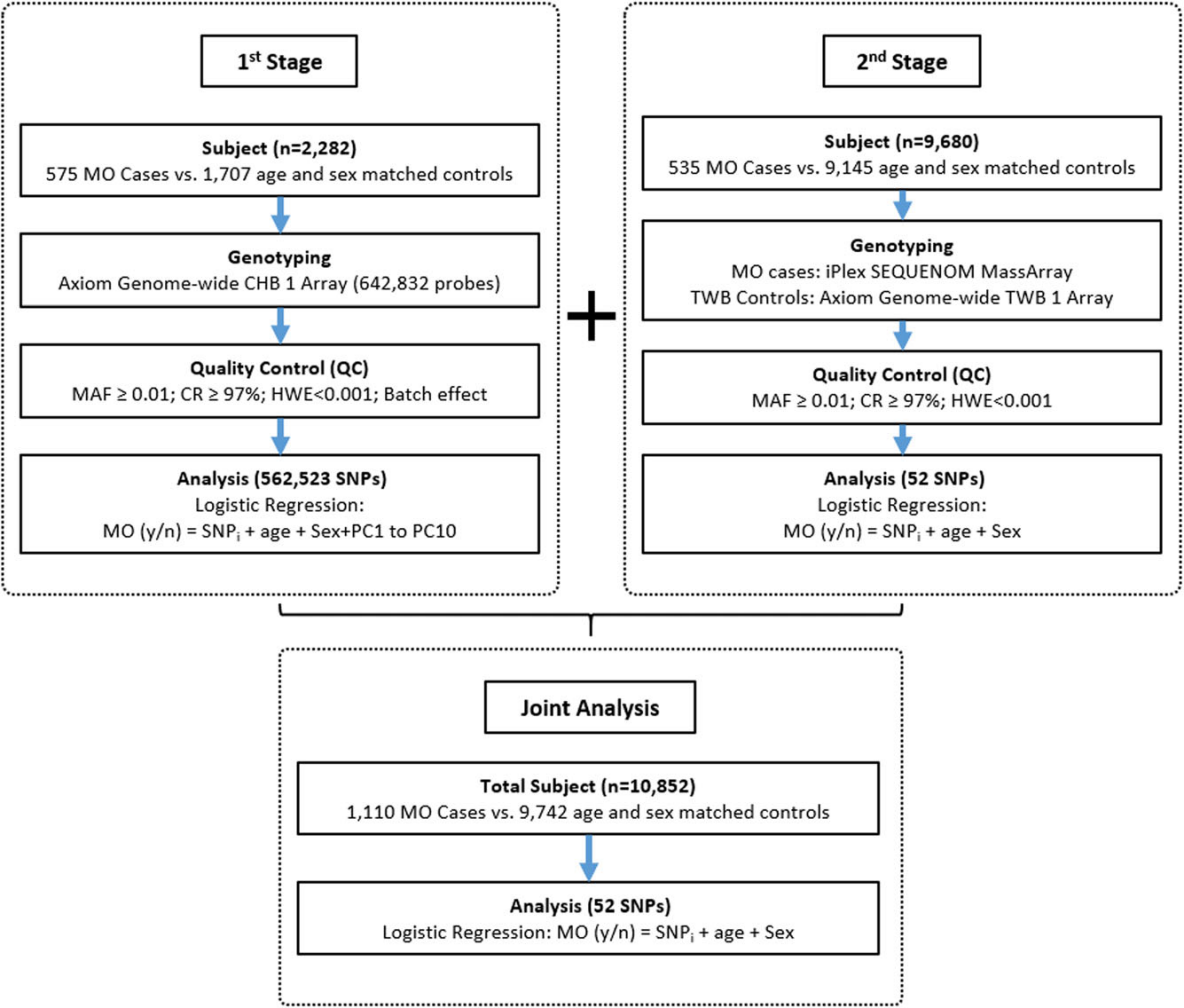
The study flow chart of two-stage GWAS

The study flow chart is provided in Fig. 2. MO cases, defined by BMI ≥ 35 kg/m^2^ [4, 5], were recruited from the Minimally Invasive Surgery Center of Min-Sheng General Hospital, Taoyuan city, Taiwan. Patients diagnosed with psychosis, developmental diseases, and cancer were excluded. In western countries, MO is defined as BMI ≥ 40 kg/m^2^. Bariatric surgery is an optional treatment for people with MO that meet the following criteria: BMI ≥ 40 kg/m^2^ or between 35 and 40 kg/m^2^ and other significant diseases (for example type 2 diabetes or high blood pressure). However, it is generally accepted that the BMI cut-off points for defining obesity should be lower for Asians [71]. In 2011, the Asian Pacific Metabolic and Bariatric Surgery Society suggested that [5] bariatric surgery should be considered as a treatment option for obesity in people with Asian ethnicity when (1) BMI > 35 kg/m^2^ with or without co-morbidities, or (2) BMI ranged from 32 to 35 kg/m^2^ with co-morbidities. We used the definition of Asian Pacific Metabolic and Bariatric Surgery Society to recruit patients with MO.

For the control groups. In the first discovery stage, 1707age (± 3 years) and sex matched controls (BMI < 35 kg/m^2^) were included from Han-Chinese Cell and Genome Bank in Taiwan (HanBKT) established from October 1, 2002, to January 14, 2004. The recruitment procedure and data collection have been previously reported [72]. In brief, it aimed to collect representative genetic samples to document genetic diversity in Taiwan Han-Chinese and to serve as controls in disease association studies. In the second confirmatory stage, another independent set of 9145age (± 5 years) and sex matched controls (BMI < 35 kg/m^2^) was included from Taiwan Biobank (TWB) [73]. Details on the TWB can be found on its official website (https://taiwanview.twbiobank.org.tw/index). Altogether, 10,852 subjects (1110 MO cases and 9742matched controls) were included in the joint GWAS.

### DNA extraction and genotyping

DNA from MO cases was extracted from buffy coats of whole blood using the phenol-chloroform method [74]. Genomic DNA of controls collected by HanBKT and TWB were isolated from leukocytes using Puregene® DNA purification kit (Gentra Systems, Minneapolis, MN, USA) [72, 73, 75] and its quality was assessed from the ratio of absorbance recorded at 260 and 280 nm wavelengths using a NanoDrop ND-1000 spectrophotometer (NanoDrop Technologies, DE, USA) [72–75]. Genotyping was carried out by the National Center for Genome Medicine (NCGM) in IBMS, AS (http://ncgm.sinica.edu.tw/ncgm_02/index.html).

In the first-stage GWAS, Affymetrix Axiom™ Genome-Wide CHB 1 Array (Thermo Fisher Scientific Inc., US) was used as the genotyping platform for both MO cases and controls. The array had 640,674 markers. The quality of genotyping was evaluated by genotype calling rate (CR), minor allele frequency (MAF), and Hardy-Weinberg Equilibrium (HWE). SNPs that failed to pass the quality control (CR < 97%, MAF < 5%, or HWE < 0.001) were excluded. The remaining 562,523 SNPs were used in the first-stage GWAS.

In the second stage, the top SNPs selected from the first stage were validated using an independent sample set, as previously described (535 MO cases and 6242 controls). For MO subjects, the SNPs were genotyped using MassARRAY® iPLEX Gold array from SEQUENOM MassARRAY® System. For the TWB controls, SNPs were genotyped by Axiom™ Genome-Wide TWB Array.

### Statistics

To search for SNPs associated with MO, logistic regression (dichotomous MO status as outcome) analysis was performed at both stages, and joint analysis was conducted after sex and age adjustment. To adjust for population stratification and batch effects, principle components (PCs) from 1 to 10 derived from the principle component analysis (PCA) were included in the regression model. We adopted an ordinal genotype coding system (number of minor allele: 0, 1, and 2). Haploview software [76] was used to analyze linkage disequilibrium (LD) structure of the identified SNPs. Data were analyzed with PLINK and SAS 9.4 (SAS Inc., NC, USA).

## Supplementary information

**Additional file 1: Table S1.** Comparison of the basic characteristics of the MO and controls between the stage 1 and stage 2. **Table S2.** The top 80 significantly associated SNPs in the first stage of the GWAS. **Table S3.** Replication study of the 85 loci associated with BMI in the Japanese population. **Table S4.** The association between the rs116917414 and BMI. **Figure. S1.** The LD plot of the FTO gene on Chr 16

## Data Availability

The data used in this study can be applied from Taiwan Biobank at https://www.twbiobank.org.tw/new_web_en/index.php.

## Abbreviations

BMI: Body mass index
GWAS: Genome-wide association study
LD: Linkage disequilibrium
MAF: Minor allele frequency
MO: Morbid obesity
SNP: Single nucleotide polymorphism
TWB: Taiwan Biobank
