# Supplementary material for: Genome-wide association study of morbid obesity in Han Chinese
Source: BMC Genet. 2019 Dec 18;20:97. doi: 10.1186/s12863-019-0797-x (PMC6921553; doi:10.1186/s12863-019-0797-x)

# **Genome-Wide Association Study of Morbid Obesity in Han Chinese**

Kuang-Mao Chiang^1^**^+^**, Heng-Cheng Chang^2^**^+^**; Hsin-Chou Yang^3^; Chien-Hsiun Chen^1^; Hsin-Hung Chen^5^; Wei-Jei Lee*^6^; Wen-Harn Pan*^1, 7^

Additional file 1

| **Table S1: Comparison of the basic characteristics of the MO and controls between the stage 1 and stage 2.** | | | | | | | | |
| --- | --- | --- | --- | --- | --- | --- | --- | --- |
|  |  | **Stage 1** | |  | **Stage 2** | |  | **p-value** |
|  |  | N=553 | |  | N=443 | |  |  |
|  |  |  |  |  |  |  |  |  |
| **MO** | Sex (No. of Male, %) | 170 | 30.70% |  | 156 | 35.20% |  | 0.051 |
|  |  |  |  |  |  |  |  |  |
|  | Age (mean, SD) | 33.7 | 7.9 |  | 31.3 | 8.5 |  | 0.07 |
|  |  |  |  |  |  |  |  |  |
|  | BMI (mean, SD) | 42.1 | 6.7 |  | 42.9 | 6.2 |  | 0.11 |
|  |  |  |  |  |  |  |  |  |
|  |  |  |  |  |  |  |  |  |
|  |  | N=1729 | |  | N=9237 | |  |  |
| **Control** | Sex (No. of Male, %) | 793 | 45.90% |  | 4444 | 49.47% |  | 0.0001 |
|  |  |  |  |  |  |  |  |  |
|  | Age (mean, SD) | 46 | 14.6 |  | 48.8 | 11.1 |  | 0.002 |
|  |  |  |  |  |  |  |  |  |
|  | BMI (mean, SD) | 24 | 4.4 |  | 24.1 | 3.6 |  | 0.21 |

| **TableS2. The Top 80 significantly associated SNPs in the first stage of the GWAS** | | | | | | |
| --- | --- | --- | --- | --- | --- | --- |
| **CHR** | **SNP** | **Position** | **Relative Genes** | **OR** | **SE** | **Raw p-value** |
| **4** | **rs116917414** | **23559886** |  | **16.37** | **0.35** | **1.56E-15** |
| **16** | **rs8050136** | **53816275** | ***FTO*** | **1.73** | **0.11** | **2.11E-07** |
| **16** | **rs9939609** | **53820527** | ***FTO*** | **1.73** | **0.11** | **2.20E-07** |
| **16** | **rs9941349** | **53825488** | ***FTO*** | **1.62** | **0.09** | **2.82E-07** |
| **2** | **rs6757087** | **212680523** | ***ERBB4*** | **1.46** | **0.08** | **5.19E-07** |
| **4** | **rs142176079** | **143467124** | ***INPP4B*** | **2.55** | **0.19** | **5.25E-07** |
| 16 | rs1121980 | 53809247 | *FTO* | 1.59 | 0.09 | 1.16E-06 |
| 16 | rs9937354 | 53799847 | *FTO* | 1.57 | 0.09 | 1.18E-06 |
| 16 | rs1421085 | 53800954 | *FTO* | 1.65 | 0.10 | 1.31E-06 |
| 11 | rs2924697 | 68586939 | *CPT1A* | 1.77 | 0.12 | 1.36E-06 |
| 2 | rs74258985 | 39367999 |  | 3.92 | 0.29 | 2.78E-06 |
| 17 | rs465563 | 3513719 | *SHPK* | 1.46 | 0.08 | 3.04E-06 |
| 14 | rs12884804 | 51853018 |  | 1.41 | 0.08 | 5.63E-06 |
| 5 | rs160936 | 55472289 | *ANKRD55* | 2.19 | 0.17 | 5.74E-06 |
| 16 | rs12925846 | 6926667 | *RBFOX1* | 0.68 | 0.09 | 6.04E-06 |
| 15 | rs8036282 | 97043297 |  | 1.62 | 0.11 | 6.54E-06 |
| 11 | rs3019613 | 68589257 | *CPT1A* | 1.69 | 0.12 | 6.71E-06 |
| 20 | rs6069477 | 54491980 |  | 1.40 | 0.08 | 9.40E-06 |
| 14 | rs11626956 | 51850031 |  | 1.40 | 0.08 | 9.57E-06 |
| 1 | rs3003482 | 17550601 | *PADI1* | 0.70 | 0.08 | 1.02E-05 |
| 1 | rs10925978 | 239960932 | *CHRM3* | 0.66 | 0.09 | 1.07E-05 |
| 3 | rs2059000 | 175200700 | *NAALADL2* | 1.62 | 0.11 | 1.15E-05 |
| 15 | rs16964412 | 32965448 | *SCG5* | 0.65 | 0.10 | 1.18E-05 |
| 2 | rs9808434 | 18694885 |  | 1.45 | 0.09 | 1.29E-05 |
| 16 | rs17235335 | 6928991 | *RBFOX1* | 0.69 | 0.09 | 1.30E-05 |
| 8 | rs16913537 | 85901857 |  | 3.48 | 0.29 | 1.36E-05 |
| 4 | rs12649045 | 122957607 |  | 0.71 | 0.08 | 1.40E-05 |
| 13 | rs4884781 | 69004278 |  | 1.48 | 0.09 | 1.53E-05 |
| 18 | rs2126015 | 65545054 | *LOC643542* | 0.70 | 0.08 | 1.76E-05 |
| 10 | rs4918398 | 110452276 |  | 1.43 | 0.08 | 1.78E-05 |
| 16 | rs72805611 | 53831354 | *FTO* | 1.54 | 0.10 | 1.93E-05 |
| 8 | rs60170826 | 80126988 |  | 1.45 | 0.09 | 1.99E-05 |
| 13 | rs9571961 | 69167161 |  | 1.48 | 0.09 | 2.05E-05 |
| 16 | rs8059403 | 6927470 | *RBFOX1* | 0.69 | 0.09 | 2.14E-05 |
| 2 | rs490805 | 80198597 | *CTNNA2* | 1.42 | 0.08 | 2.22E-05 |
| 3 | rs59778278 | 120809596 | *STXBP5L* | 0.53 | 0.15 | 2.31E-05 |
| 13 | rs9541480 | 69222120 |  | 1.47 | 0.09 | 2.34E-05 |
| 8 | rs3104966 | 96594034 | *LOC100616530* | 1.46 | 0.09 | 2.59E-05 |
| 13 | rs10711827 | 69220148 |  | 1.47 | 0.09 | 2.62E-05 |
| 12 | rs799575 | 58069107 |  | 0.72 | 0.08 | 2.74E-05 |
| 5 | rs55696513 | 115733851 | *LOC101927212* | 0.72 | 0.08 | 2.78E-05 |
| 1 | rs12408734 | 239952474 | *CHRM3* | 0.72 | 0.08 | 2.86E-05 |
| 15 | rs8037286 | 97036152 |  | 1.58 | 0.11 | 2.92E-05 |
| 11 | rs3019587 | 68569642 | *CPT1A* | 1.80 | 0.14 | 2.97E-05 |
| 9 | rs59589985 | 18652065 | *ADAMTSL1* | 0.50 | 0.17 | 2.98E-05 |
| 4 | rs116942732 | 89126071 | *ABCG2* | 2.21 | 0.19 | 2.98E-05 |
| 13 | rs9571975 | 69218765 |  | 1.47 | 0.09 | 3.02E-05 |
| 6 | rs10485084 | 6781726 |  | 0.64 | 0.11 | 3.21E-05 |
| 1 | rs12563366 | 237796850 | *RYR2* | 0.71 | 0.08 | 3.24E-05 |
| 3 | rs5850130 | 73735845 |  | 1.72 | 0.13 | 3.25E-05 |
| 6 | rs7743432 | 151912507 | *CCDC170* | 1.47 | 0.09 | 3.26E-05 |
| 13 | rs17083340 | 68989546 |  | 1.45 | 0.09 | 3.28E-05 |
| 2 | rs2892923 | 165438473 | *GRB14* | 1.67 | 0.12 | 3.38E-05 |
| 17 | rs11869824 | 66753420 |  | 1.51 | 0.10 | 3.46E-05 |
| 18 | rs9959475 | 9009311 |  | 0.72 | 0.08 | 3.54E-05 |
| 2 | rs17018359 | 80216176 | *CTNNA2* | 1.42 | 0.08 | 3.60E-05 |
| 7 | rs6460030 | 72207443 | *TYW1B* | 1.37 | 0.08 | 3.79E-05 |
| 13 | rs735600 | 69002290 |  | 1.43 | 0.10 | 3.81E-05 |
| 1 | rs544700 | 38559533 |  | 1.40 | 0.09 | 4.11E-05 |
| 2 | rs4146084 | 126299170 |  | 1.58 | 0.08 | 4.22E-05 |
| 5 | rs34971 | 115795188 | *SEMA6A/LOC101927233* | 1.36 | 0.11 | 4.25E-05 |
| 8 | rs117364719 | 36954829 |  | 2.24 | 0.08 | 4.45E-05 |
| 8 | rs73343004 | 76345695 |  | 2.36 | 0.20 | 4.57E-05 |
| 19 | rs2053079 | 30987423 | *ZNF536* | 0.66 | 0.21 | 4.61E-05 |
| 4 | rs12644266 | 184830847 | *STOX2* | 1.36 | 0.10 | 4.62E-05 |
| 7 | rs2909969 | 71982924 |  | 1.35 | 0.08 | 4.71E-05 |
| 8 | rs61470037 | 130467803 |  | 0.63 | 0.07 | 4.75E-05 |
| 2 | rs17039237 | 1984653 | *MYT1L* | 0.72 | 0.11 | 4.76E-05 |
| 13 | rs2875555 | 69002447 |  | 1.43 | 0.08 | 4.78E-05 |
| 8 | rs3104994 | 96571283 | *LOC100616530* | 1.54 | 0.09 | 4.84E-05 |
| 4 | rs17694759 | 122938545 |  | 0.73 | 0.11 | 4.86E-05 |
| 16 | rs11642841 | 53845487 | *FTO* | 2.08 | 0.08 | 4.90E-05 |
| 5 | rs26592 | 115775542 |  | 1.37 | 0.18 | 4.91E-05 |
| 8 | rs72621457 | 64548431 |  | 0.70 | 0.08 | 5.12E-05 |
| 8 | rs16937550 | 72191285 | *EYA1* | 0.73 | 0.09 | 5.15E-05 |
| 18 | rs76010193 | 65526327 | *LOC643542* | 0.73 | 0.08 | 5.21E-05 |
| 18 | rs34573570 | 944492 |  | 1.52 | 0.08 | 5.26E-05 |
| 4 | rs10007118 | 38382277 |  | 1.64 | 0.10 | 5.27E-05 |
| 13 | rs55824229 | 68989577 |  | 1.45 | 0.12 | 5.41E-05 |
| 9 | rs2150334 | 27356691 | *MOB3B* | 0.72 | 0.09 | 5.42E-05 |
|  |  |  |  |  |  |  |

| **Table S3. Replication study of the 85 loci associated with BMI in the Japanese population.** | | | | | | | | |  |
| --- | --- | --- | --- | --- | --- | --- | --- | --- | --- |
| **SNP** | **Chr.** | **Position (bp)** | **Alleles REF/ALT** | **Positional** |  | **Meta-analysis (n=173430)** |  | **MO-GWAS** |  |
|  |  |  |  | **candidate genes** |  | **P value** |  | **p-value** |  |
| **rs80234489** | **12** | **31,441,179** | **A/C** | ***FAM60A*** |  | ***1.05E-11*** |  | ***0.00437*** |  |
| **rs11602339** | **11** | **47,761,471** | **C/T** | ***FNBP4*** |  | ***1.01E-09*** |  | ***0.00952*** |  |
| **rs3932549** | **9** | **97,073,588** | **A/C** | ***ZNF169,NUTM2F*** |  | ***1.97E-09*** |  | ***0.02778*** |  |
| **rs2540034** | **16** | **4,022,694** | **C/T** | ***ADCY9*** |  | ***2.97E-12*** |  | ***0.02818*** |  |
| **rs1190736** | **X** | **136,113,464** | **C/A** | ***GPR101*** |  | ***1.31E-08*** |  | ***0.0424*** |  |
| **rs4357030** | **5** | **124,316,031** | **C/T** | ***ZNF608,LOC101927421*** |  | ***3.60E-10*** |  | ***0.04582*** |  |
| rs4686392 | 3 | 185,524,081 | A/G | *IGF2BP2* |  | 1.42E-18 |  | 0.05114 |  |
| rs2495707 | 10 | 102,425,949 | A/G | *HIF1AN,PAX2* |  | 1.21E-09 |  | 0.07167 |  |
| rs329120 | 5 | 133,861,756 | C/T | *JADE2* |  | 3.70E-08 |  | 0.07583 |  |
| rs7903146 | 10 | 114,758,349 | C/T | *TCF7L2* |  | 9.41E-12 |  | 0.08142 |  |
| rs1568079 | 10 | 125,251,751 | T/A | *BUB3,GPR26* |  | 1.80E-12 |  | 0.1038 |  |
| rs11030100 | 11 | 27,677,586 | G/T | *BDNF* |  | 1.19E-28 |  | 0.1248 |  |
| rs143665886 | 7 | 115,368,366 | T/C | *LINC01392,TFEC* |  | 9.46E-09 |  | 0.1323 |  |
| rs9568867 | 13 | 54,107,352 | G/A | *LINC01065,LINC00558* |  | 1.03E-14 |  | 0.1353 |  |
| rs939584 | 2 | 621,558 | C/T | *FAM150B,TMEM18* |  | 6.40E-23 |  | 0.1364 |  |
| rs77636220 | 8 | 64,552,779 | G/A | *LOC102724612,LINC01289* |  | 2.80E-11 |  | 0.1386 |  |
| rs2574704 | 3 | 11,655,381 | T/C | *VGLL4* |  | 4.70E-08 |  | 0.1594 |  |
| rs633715 | 1 | 177,852,580 | T/C | *LOC101928778,SEC16B* |  | 1.05E-33 |  | 0.1704 |  |
| rs11642015 | 16 | 53,802,494 | C/T | *FTO* |  | 2.04E-81 |  | 0.1755 |  |
| rs10868215 | 9 | 87,234,111 | T/C | *SLC28A3,NTRK2* |  | 1.34E-08 |  | 0.2188 |  |
| rs6913361 | 6 | 34,179,390 | A/G | *GRM4,HMGA1* |  | 2.73E-14 |  | 0.2257 |  |
| rs1379871 | X | 31,854,782 | G/C | *DMD* |  | 1.05E-08 |  | 0.226 |  |
| rs5945324 | X | 152,894,551 | G/C | *FAM58A,DUSP9* |  | 1.33E-11 |  | 0.2391 |  |
| rs2076463 | 1 | 27,971,092 | A/G | *FGR,IFI6* |  | 1.68E-08 |  | 0.244 |  |
| rs77511173 | 12 | 53,883,537 | T/C | *MAP3K12* |  | 1.46E-08 |  | 0.2686 |  |
| rs491055 | 1 | 190,308,834 | A/G | *BRINP3* |  | 2.82E-10 |  | 0.2714 |  |
| rs12617004 | 2 | 142,615,136 | G/C | *LRP1B* |  | 5.83E-09 |  | 0.2782 |  |
| rs148546399 | 6 | 64,705,610 | G/A | *EYS* |  | 1.13E-09 |  | 0.3138 |  |
| rs16978956 | 20 | 18,288,165 | A/G | *ZNF133* |  | 3.46E-09 |  | 0.327 |  |
| rs75766425 | 14 | 52,511,911 | G/C | *NID2* |  | 1.28E-10 |  | 0.348 |  |
| rs28857569 | 8 | 76,697,034 | T/C | *HNF4G,LINC01111* |  | 2.00E-09 |  | 0.3496 |  |
| rs8192473 | 3 | 42,299,399 | C/T | *CCK* |  | 3.58E-09 |  | 0.3531 |  |
| rs62034325 | 16 | 28,538,640 | A/G | *IL27,NUPR1* |  | 3.81E-10 |  | 0.3543 |  |
| rs7305242 | 12 | 112,256,762 | T/C | *ALDH2,MAPKAPK5-AS1* |  | 2.21E-08 |  | 0.3968 |  |
| rs10062657 | 5 | 95,867,908 | C/A | *PCSK1* |  | 3.89E-25 |  | 0.3998 |  |
| rs3121672 | X | 117,916,370 | T/C | *IL13RA1* |  | 2.90E-17 |  | 0.429 |  |
| rs6881648 | 5 | 74,991,849 | A/C | *POC5* |  | 2.03E-12 |  | 0.4367 |  |
| rs12569457 | 10 | 99,096,676 | C/T | *FRAT2,RRP12* |  | 6.67E-09 |  | 0.4397 |  |
| rs9983113 | 21 | 40,315,316 | G/T | *LOC400867,LOC101928435* |  | 2.32E-08 |  | 0.4471 |  |
| rs2247627 | 20 | 54,145,086 | G/A | *LINC01441,CBLN4* |  | 2.54E-08 |  | 0.4698 |  |
| rs35261542 | 6 | 20,675,792 | C/A | *CDKAL1* |  | 3.61E-29 |  | 0.4718 |  |
| rs713586 | 2 | 25,158,008 | T/C | *ADCY3,DNAJC27* |  | 4.92E-13 |  | 0.477 |  |
| rs10197655 | 2 | 58,791,420 | G/A | *LINC01122* |  | 3.63E-09 |  | 0.5193 |  |
| rs1518170 | 18 | 40,708,905 | T/C | *RIT2,SYT4* |  | 6.80E-09 |  | 0.5263 |  |
| rs10174398 | 2 | 51,195,601 | T/C | *NRXN1* |  | 4.65E-09 |  | 0.5359 |  |
| rs4788694 | 16 | 73,070,083 | C/G | *ZFHX3* |  | 2.54E-08 |  | 0.5419 |  |
| rs72749754 | 15 | 62,319,432 | G/C | *VPS13C* |  | 7.68E-10 |  | 0.5518 |  |
| rs7912454 | 10 | 18,584,792 | A/G | *CACNB2* |  | 3.62E-10 |  | 0.5663 |  |
| rs2593235 | 15 | 57,541,201 | G/A | *TCF12* |  | 1.60E-08 |  | 0.5757 |  |
| rs1035491 | 5 | 63,962,177 | A/G | *RGS7BP,FAM159B* |  | 5.63E-09 |  | 0.5907 |  |
| rs183975233 | 6 | 32,437,160 | T/A | *HLA-DRA,HLA-DRB5* |  | 7.51E-16 |  | 0.6257 |  |
| rs6947395 | 7 | 69,406,661 | A/T | *AUTS2* |  | 4.81E-12 |  | 0.6306 |  |
| rs1846974 | 5 | 87,969,927 | G/A | *LINC00461* |  | 1.81E-15 |  | 0.6341 |  |
| rs11130319 | 3 | 52,755,592 | A/T | *NEK4* |  | 3.72E-11 |  | 0.645 |  |
| rs139913 | 22 | 40,713,861 | T/A | *TNRC6B* |  | 2.29E-15 |  | 0.6502 |  |
| rs10208649 | 2 | 54,161,363 | T/C | *PSME4* |  | 4.95E-12 |  | 0.6511 |  |
| rs3205718 | 12 | 50,261,809 | C/T | *FAIM2* |  | 4.62E-10 |  | 0.6527 |  |
| rs77489951 | 2 | 38,750,287 | C/T | *LOC101929596,HNRNPLL* |  | 9.39E-09 |  | 0.6592 |  |
| rs729050 | 14 | 94,109,502 | G/T | *UNC79* |  | 2.08E-09 |  | 0.6723 |  |
| rs5015933 | 9 | 128,137,418 | T/C | *GAPVD1,MAPKAP1* |  | 6.12E-10 |  | 0.6881 |  |
| rs7020996 | 9 | 22,129,579 | C/T | *CDKN2B-AS1,DMRTA1* |  | 5.87E-18 |  | 0.6939 |  |
| rs6734118 | 2 | 37,559,355 | C/A | *PRKD3,QPCT* |  | 3.42E-12 |  | 0.7057 |  |
| rs6433857 | 2 | 181,517,996 | C/T | *CWC22,SCHLAP1* |  | 4.57E-08 |  | 0.729 |  |
| rs111612372 | 2 | 20,433,218 | T/C | *SDC1,PUM2* |  | 2.33E-08 |  | 0.7444 |  |
| rs4366055 | 8 | 95,507,328 | A/C | *KIAA1429* |  | 2.12E-09 |  | 0.7472 |  |
| rs10795945 | 10 | 12,302,607 | T/C | *CDC123,CAMK1D* |  | 1.10E-09 |  | 0.7569 |  |
| rs1832886 | 10 | 94,477,539 | G/A | *HHEX,EXOC6* |  | 2.59E-12 |  | 0.7641 |  |
| rs1996023 | 4 | 45,164,637 | T/G | *GNPDA2,GABRG1* |  | 1.06E-17 |  | 0.7835 |  |
| rs180950758 | 17 | 29,036,425 | A/T | *SUZ12P1* |  | 2.63E-08 |  | 0.7982 |  |
| rs860295 | 1 | 155,767,708 | A/G | *GON4L* |  | 8.66E-11 |  | 0.8038 |  |
| rs6567160 | 18 | 57,829,135 | T/C | *PMAIP1,MC4R* |  | 8.44E-37 |  | 0.8043 |  |
| rs9397585 | 6 | 153,396,875 | T/C | *RGS17* |  | 5.05E-09 |  | 0.8156 |  |
| rs2390669 | 2 | 169,091,942 | A/C | *STK39* |  | 5.63E-10 |  | 0.8159 |  |
| rs80117551 | 10 | 69,834,828 | C/T | *HERC4* |  | 1.57E-08 |  | 0.83 |  |
| rs6089584 | 20 | 60,564,086 | G/C | *TAF4* |  | 3.58E-09 |  | 0.8319 |  |
| rs4308481 | 5 | 122,652,106 | C/T | *PRDM6,CEP120* |  | 9.71E-09 |  | 0.8594 |  |
| rs4409766 | 10 | 104,616,663 | T/C | *C10orf32-ASMT* |  | 5.08E-11 |  | 0.8737 |  |
| rs4790981 | 17 | 65,921,834 | A/G | *BPTF* |  | 4.65E-10 |  | 0.9035 |  |
| rs6529684 | X | 53,542,107 | A/G | *HSD17B10,HUWE1* |  | 2.78E-08 |  | 0.9131 |  |
| rs16937956 | 11 | 8,404,501 | A/G | *LMO1,STK33* |  | 5.16E-11 |  | 0.914 |  |
| rs60808706 | 11 | 2,857,233 | G/A | *KCNQ1* |  | 1.24E-38 |  | 0.9191 |  |
| rs2206271 | 6 | 50,786,008 | T/A | *TFAP2B* |  | 2.77E-18 |  | 0.9296 |  |
| rs12597682 | 16 | 20,258,432 | C/A | *GPR139,GP2* |  | 8.25E-12 |  | 0.9375 |  |
| rs1907240 | 10 | 122,897,959 | G/A | *MIR5694,FGFR2* |  | 3.47E-11 |  | 0.9708 |  |
| rs35560038 | 19 | 46,175,046 | A/T | *GIPR* |  | 2.83E-52 |  | 0.9836 |  |
|  |  |  |  |  |  |  |  |  |  |

| Table S4. The association between the rs116917414 and BMI | | | |  |
| --- | --- | --- | --- | --- |
| **rs116917414** | **Beta** | **SE** | **t value** | **p-value** |
| G/A vs. G/G | -0.13 | 0.3 | -0.43 | 0.67 |
| A/A vs. G/G | 1.06 | 1.6 | 0.65 | 0.51 |
| Sample size: GG:GA:AA=1281:159:5; Model: BMI~Genotype + SEX | | | | |

|  |
| --- |

Figure S1. The LD plot of the FTO gene on Chr 16.


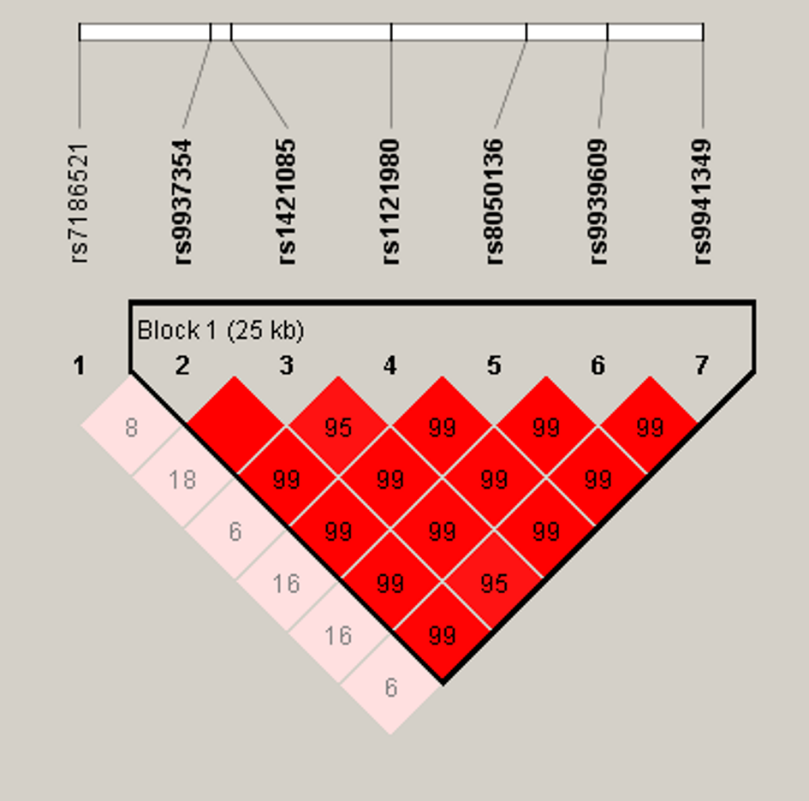

Supplement: Supplementary file 1 — Additional file 1: Table S1. Comparison of the basic characteristics of the MO and controls between the stage 1 and stage 2. Table S2. The top 80 significantly associated SNPs in the first stage of the GWAS. Table S3. Replication study of the 85 loci associated with BMI in the Japanese population. Table S4. The association between the rs116917414 and BMI. Figure. S1. The LD plot of the FTO gene on Chr 16 [file 12863_2019_797_MOESM1_ESM.docx]
